# Accurate 3D Shape, Displacement and Deformation Measurement Using a Smartphone

**DOI:** 10.3390/s19030719

**Published:** 2019-02-10

**Authors:** Liping Yu, Ran Tao, Gilles Lubineau

**Affiliations:** COHMAS Laboratory, Physical Sciences and Engineering Division (PSE), King Abdullah University of Science and Technology (KAUST), Thuwal 23955-6900, Saudi Arabia; ran.tao@kaust.edu.sa

**Keywords:** stereo-digital image correlation, smartphone, optical attachment

## Abstract

The stereo-digital image correlation technique using two synchronized industrial-grade cameras has been extensively used for full-field 3D shape, displacement and deformation measurements. However, its use in resource-limited institutions and field settings is inhibited by the need for relatively expensive, bulky and complicated experimental set-ups. To mitigate this problem, we established a cost-effective and ultra-portable smartphone-based stereo-digital image correlation system, which only uses a smartphone and an optical attachment. This optical attachment is composed of four planar mirrors and a 3D-printed mirror support, and can split the incoming scene into two sub-images, simulating a stereovision system using two virtual smartphones. Although such a mirror-based system has already been used for stereo-image correlation, this is the first time it has been combined with a commercial smartphone. This publication explores the potential and limitations of such a configuration. We first verified the effectiveness and accuracy of this system in 3D shape and displacement measurement through shape measurement and in-plane and out-of-plane translation tests. Severe thermal-induced virtual strains (up to 15,000 με) were found in the measured results due to the smartphone heating. The mechanism for the generation of the temperature-dependent errors in this system was clearly and reasonably explained. After a simple preheating process, the smartphone-based system was demonstrated to be accurate in measuring the strain on the surface of a loaded composite specimen, with comparable accuracy to a strain gauge. Measurements of 3D deformation are illustrated by tracking the deformation on the surface of a deflating ball. This cost-effective and ultra-portable smartphone-based system not only greatly decreases the hardware investment in the system construction, but also increases convenience and efficiency of 3D deformation measurements, thus demonstrating a large potential in resource-limited and field settings.

## 1. Introduction

Optical methods for 3D shape, displacement and deformation measurements of materials and structures under various loadings allow for the visualization of surface profile change and the quantification of surface deformation evolution, significantly advancing our knowledge of material properties and their failure mechanisms. These optical methods mainly include interferometric techniques, such as electronic speckle pattern interferometry [1], digital shearography [2], and holographic interferometry [3,4], and non-interferometric techniques, such as stereo-digital image correlation [5,6,7] (stereo-DIC, also termed as 3D-DIC). Among these methods, stereo-DIC offers several distinct advantages over the other interferometric techniques, such as its simple optical arrangement, easy specimen preparation, minimal requirements for the experimental environment and a wide range of applicability with adjustable spatial resolution. Thus, stereo-DIC has become a popular and powerful modern technique in 3D shape, displacement and deformation measurements [8].

However, the requirement of two industrial-grade cameras and a synchronization unit usually makes the construction of a regular binocular stereo-DIC system both expensive and complicated. Moreover, a commercial stereo-DIC system is costly for resource-limited institutions and for professionals in other fields who have demands in 3D deformation measurements. To mitigate this problem, various single-camera stereovision techniques, including mirror-based [9,10,11,12], prism-based [13,14], and diffraction grating-based [15,16] approaches, have been developed by incorporating a set of planar mirrors, a prism or a diffraction grating, and have been shown to be effective in 3D shape and deformation measurements. With the aid of these additional optical devices, the incoming light rays are equally split into two sub-images or diffracted into two diffraction images projected at different angles on the camera sensor. The sub-images could be considered as a pseudo-stereo image pair recorded from two different orientations by two virtual cameras. More recently, a novel color stereo-DIC method [17,18] using a single three charge-coupled device (3CCD) or a complementary metal-oxide semiconductor (CMOS) color camera was proposed for full-frame 3D shape and deformation measurements. This approach enables two images of the object surface to overlap each other via different color channels, which ensures the full application of the spatial resolution of the camera sensor for obtaining 3D information. Indeed, the above-mentioned research and efforts avoid the synchronization problem and halve the cost of cameras, but they still require an industrial-grade camera, an image acquisition computer and a relatively bulky and complex optical auxiliary device. As such, the translation of these existing two-camera or single-camera stereo-DIC systems to cost-effective, easy-to-use and field-portable instruments may open up a large number of new applications, and would also positively impact research and educational efforts in developing countries and resource-limited institutions, helping the popularization of advanced scientific instruments and measurement tools.

For this broad purpose, consumer electronics devices, such as digital single-lens reflex (DSLR) camera [19] and infrared Kinect sensors [20], have been emerging as effective instruments to create cost-effective, portable and readily accessible alternatives to some of the commercial 3D shape and deformation measuring systems. Compared with the above-mentioned consumer electronics devices, smartphones are more attractive in establishing a stereo-DIC system as they are more portable and almost ubiquitous. More importantly, smartphones have experienced massive advances in their hardware system in recent years, providing users with high-end components including high-speed and high-resolution digital cameras, graphics processing units (GPUs), and various sensors (e.g., gravity sensor, acceleration sensor, gyroscope and GPS). For example, the standard resolution for the back camera in popular smartphones is usually larger than one megapixel, and the maximum frame rate of the state-of-the-art smartphones (e.g., Samsung S9, Sony Xperia and Huawei P20) can reach 960 frames per second. This superior performance makes smartphones an ideal platform for conducting various scientific research and opens up the opportunity for researchers in resource-limited settings to satisfy the demand for cost-effective and field-portable measurement tools [21]. Due to their distinct advantages, smartphones have become versatile platforms for a wide range of applications in bioscience and biomedical imaging, including DNA imaging and sizing [22], microscopic visualization [23,24], imaging-based sensing [25,26], and diagnostics [27,28]. Also, smartphones have been increasingly used in engineering fields, such as flow visualization and quantitative velocity measurement [29,30], structural displacement monitoring [31], vibrating characteristics estimation of beams [32] and in-plane displacement and strain measurements [33]. Despite all of these recent advances and progress, full-field 3D shape, displacement and deformation measurements using a smartphone has not been reported. We propose to investigate here the potential and limitations of smartphone-based measuring systems. Advances in this field would also bring a novel functionality to smartphones (strain measurement) that can open the door to many new applications and user experiences well beyond the traditional field of mechanics. 

Here, we report the demonstration of smartphone-based 3D shape, displacement and deformation measurements using a field-portable and cost-effective optical attachment that is created with 3D printing and integrated onto the existing camera module of a smartphone. This optical attachment, which is mainly composed of four planar mirrors and a structural support, can split the incoming light rays into two parts and then direct these rays to the left and right halves of a camera sensor. By processing the left and right images using a regular stereo-DIC algorithm, full-field 3D shape, displacement and deformation measurements on the object surface can be retrieved. The accuracy and effectiveness of the established smartphone-based stereo-DIC system are first validated by shape measurements of a cylinder and a curved surface, and in-plane and out-of-plane translations of a flat plate. Then, the stability of the smartphone-based stereo-DIC system is investigated under significant self-heating of the smartphone. The mechanism for the generation of these errors is explained. Finally, we explore the feasibility of a smartphone-based stereo-DIC system through a tensile test of carbon fiber-reinforced plastics (CFRP) specimens and the 3D deformation measurement of a deflating ball after a simple preheating process.

## 2. Method

### 2.1. Experimental Set-Up

**Figure 1:**
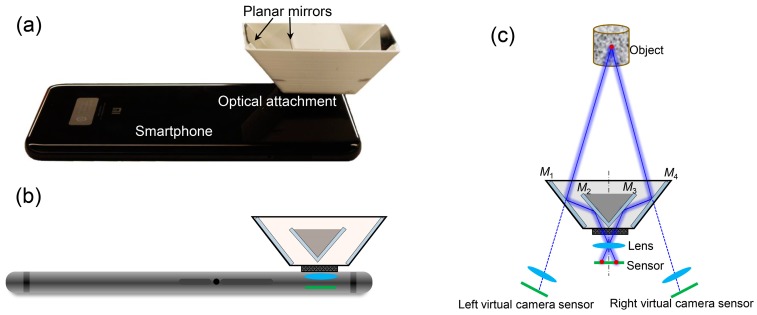
(**a**) Photograph and (**b**) schematic diagram of the established smartphone-based stereo-digital image correlation (DIC) system. (**c**) Schematic diagram of the optical paths of the smartphone-based stereo-DIC system. *M*_1_, *M*_2_, *M*_3_ and *M*_4_ represent four planar mirrors installed in the optical attachment.

Figure 1a,b show a photograph and the schematic diagram of the smartphone-based stereo-DIC system, respectively. This system only consists of a single ubiquitous smartphone and an optical attachment, which is mainly composed of four planar mirrors (denoted as *M*_1_, *M*_2_, *M*_3_ and *M*_4_) and a 3D-printed mirror support. As shown in Figure 1, the two interior mirrors (*M*_2_ and *M*_3_, 30 mm × 20 mm × 1 mm, front surface reflection) are fixed on two sides of the mirror support and form a 90° angle with each other, while two exterior mirrors (*M*_1_ and *M*_4_) are glued tightly on the outside support with a pre-estimated angle (50°) and size (30 mm × 25 mm × 1 mm, front surface reflection). The optical attachment can be connected with the smartphone with a clamp. With the aid of this optical attachment, two views of the test object surface via different optical reflection paths are projected onto two halves of the smartphone sensor, as shown in Figure 1c. The location and orientation of these two views on the recorded image can also be tuned by redesigning the mirror support. It should be noted that there are some commercially available optical attachments online, but they may not be suitable for all smartphone cameras and may suffer from the problem of refraction error caused by the rear surface reflection.

### 2.2. Principles: 3D Shape, Displacement and Deformation Measurement

**Figure 2:**
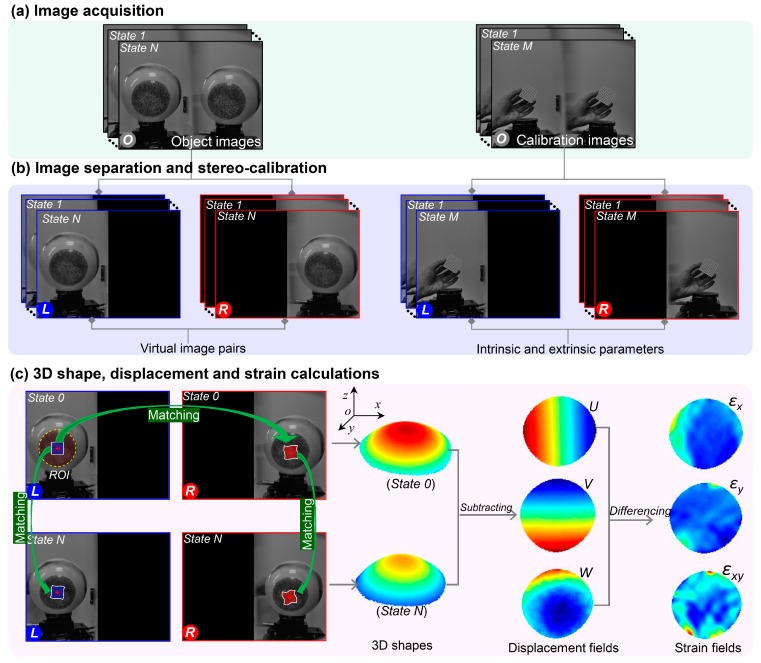
Schematic showing the procedures for measuring the 3D shape, displacement and deformation on the object surface: (**a**) image acquisition process, (**b**) image separation and the stereo-calibration process, and (**c**) the 3D shape, displacement and deformation calculation process. The letters *O*, *L* and *R* in the images represent the original image, virtual left image and virtual right sub-images, respectively. State *N* and state *M* mean the object and calibration images recorded in a certain state.

Figure 2 schematically presents the procedures to measure the 3D shape, displacement and deformation on the object surface, which is mainly based on the regular stereo-DIC method. For brevity, the principle of the method is briefly described here, and more details on the stereo-calibration and stereo-matching can be found in various references [6,7,34]. As shown in Figure 2, the procedures are separated into three steps: (a) image acquisition (including the object images and calibration images) process, (b) image separation and the stereo-calibration process, and (c) the 3D shape, displacement and deformation calculation process. As shown in Figure 2a, surface images of the test object at different configurations, which all contain two views of the object surface, are first captured by the smartphone during the test. Then, a series of images of a planar calibration target with regularly spaced circular dots are captured through a translation and/or rotation process, and used to define the world coordinate system.

After that, these recorded images, including the object images and the calibration images, are separated into left and right sub-images. As shown in Figure 2b, these sub-images merely contain a single left or right view of the object surface or calibration target, and can be seen as the virtual image pairs recorded by a virtual stereo-DIC system. With the left and right calibration images, the intrinsic and extrinsic parameters of the virtual smartphone-based stereo-DIC system can be solved through a regular stereo-calibration process, while the left and right object images, i.e., the virtual image pairs, are then used to calculate the disparity data (i.e., the differences in image coordinates between the two projection points) required for the 3D shape reconstruction. 

To accurately measure the 3D shape, displacement and deformation on the object surface, the regular stereo-DIC technique first aims to recover the 3D coordinates of a point on the object surface with respect to a world coordinate system, as shown in Figure 2c. By matching the left and right object images of the initial state (state 0), the desired disparity data of the initial state are obtained. With these disparity data, together with the previously determined calibration parameters, the 3D shapes of the region of interest (ROI) at the initial state (state 0) are retrieved according to the triangulation principle. Similarly, the profile of the ROI after deformation (state N) can also be reconstructed. By subtracting the 3D coordinates of the deformed stated (state N) from those of the initial state (state 0), 3D full-field displacement fields of different deformed state are retrieved. Finally, the full-field strain maps of the deformed state are calculated by differentiating the displacement fields using a point-wise least square strain estimation approach. Note that, except for the commercial software for DIC analysis, there are also some open-source 2D-DIC [35] and stereo-DIC codes [36,37] online, which may be very helpful for those interested in 3D shape and deformation measurement in other fields.

## 3. Experiments

**Figure 3:**
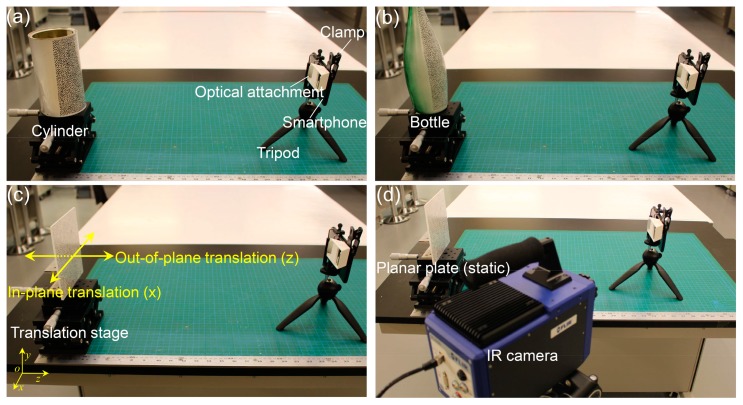
Experimental set-up for validating the effectiveness, accuracy and stability of the established smartphone-based stereo-DIC system: (**a**,**b**) Shape measurements of the regular cylinder and bottle surfaces, (**c**) in-plane and out-of-plane displacement measurements of a translated planar plate, and (**d**) a static test with the imaging system and planar plate both kept static.

To validate the effectiveness, accuracy and stability of the established smartphone-based stereo-DIC system, a series of experiments, including shape measurements of a regular cylinder and a bottle surfaces, in-plane and out-of-plane displacement measurements of a translated planar plate, and static tests, were first performed. Figure 3 shows the experimental set-up for these validation tests. As shown in Figure 3, the smartphone-based stereo-DIC system is mainly composed of an Android-based smartphone (Mi8, Xiaomi, Inc., Beijing, China), a homemade optical attachment (the adopted mirror reflects on the front surface, is 1 mm in thickness, and was bought online), and a small tripod (Type 258, Yunteng, Inc., Zhongshan, China). This smartphone adopts a Sony IMX363 sensor (4032 × 3024 pixels), and the pixel size was reported as 1.4 μm. The total price of this system is about 400 US dollars, of which the smartphone account for the vast majority: i.e., about 390 US dollars. As almost everyone has a smartphone, the actual expense of this system can be ignored. Compared with the conventional two-camera or single-camera stereo-DIC systems, this smartphone-based stereo-DIC system is ultra-portable and extremely cost-effective. During the test, only one back camera (*f*/2.4) of the smartphone and the built-in capture software (manual mode) were used for the image acquisition. Note that the resolution of the camera was preset to 2016 × 1512 pixels to speed up the calculation process. The details of the three experiments are as follows:

(1) 3D shape measurement (Figure 3a,b): In the shape measurement experiments, a cylinder with a nominal diameter of 100.20 mm (measured using a Vernier caliper) and a glass bottle with a curved surface were chosen as the test objects. Prior to the experiments, the test specimens were first sprayed using white spray paints to generate white backgrounds, and then random speckle patterns were decorated onto the background using a black marker pen. As shown in Figure 3a,b, the test object was placed well in front of the established smartphone system with an object distance measured as about 400 mm. During the test, one image of the cylinder surface, one image of the bottle surface, and 30 calibration images were captured. 

(2) 3D displacement measurement (Figure 3c): In this test, in-plane (*X* direction) and out-of-plane (*Z* direction) rigid-body translations of a flat plate (150 mm × 150 mm × 5 mm) were performed using a 2-axis translation stage with a positioning accuracy of 10 μm. The distance between the test plate and the smartphone system is also about 400 mm. During the translation tests, the plate was tightly attached to the 2-axis translation stage and first translated along the *X* direction and then the *Z* direction from −5 to 5 mm, with an increment of 1 mm between consecutive positions. For each in-plane or out-of-plane translation, an image was captured. Note that the smartphone was preheated for 45 min to eliminate the errors due to camera warm-up effect, which will be carefully studied later. 

(3) Static tests (Figure 3d): In this experiment, the indoor temperature was kept at a constant value and the planar plate was kept static. Then, surface images of the plate were automatically recorded by the smartphone every 30 s with the help of an app called Tasker (the smartphone was rooted to emulate touch input and then could be programmatically controlled by the app). The image recording process lasted for 2 h, and in total about 240 images were recorded in the test. The first recorded image was adopted as the reference image, and the remaining images were considered as the deformed images. As no external loading was applied to the plate, the actual displacements and strains on the glass plate should be zero and the measured displacements and strains can only be attributed to the instability of the system, such as the slight structural variation due to the smartphone warm-up effect. To monitor the temperature variation of the smartphone system during the test, an infrared thermal camera (SC7900, FLIR) with a resolution of 320 × 256 pixels was placed beside the smartphone stereo-DIC system to measure the full-field temperature variations of the smartphone back surface. 

## 4. Results

### 4.1. Shape Measurements of Cylinder and Curved Bottle Surfaces

During the calculation, the calibration images and images of the test objects were first exported to the computer and then processed to obtain the desired 3D information. The calibration images were used to determine the camera parameters of the smartphone-based stereo-DIC system. Table 1 lists the calibrated intrinsic and extrinsic parameters of the stereo-DIC system using two virtual smartphone cameras, where (*c_x_*, *c_y_*) are the image coordinates of the principal point, *f_x_* and *f_y_* are the focal lengths in two directions, *k*_1_ is the first-order radial distortion coefficient of the smartphone lens, (*α*, *β*, *γ*) are relative angles between the two virtual cameras and (*t_x_*, *t_y_*, *t_z_*) are the translation of right camera relative to left camera. Note that we verified that the classic radial distortion can still be applied by conducting in-plane translation tests with only the smartphone and 2D-DIC used. The reprojection error is estimated as 0.082 pixels. Then, a region of interest (ROI) was selected from the left image of the test object as the calculation region. By matching the left and right object images using the DIC algorithm, the desired disparity data of the calibration points in ROI were obtained. Finally, the 3D coordinates of these calculation points were reconstructed based on the calibrated camera parameters and disparity data according to the triangulation principle. 

**Figure 4:**
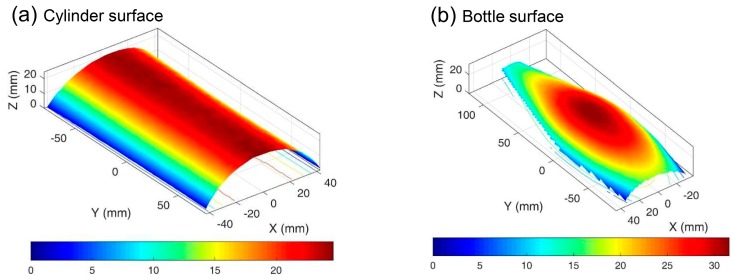
The reconstructed 3D shapes of (**a**) the cylinder and (**b**) the bottle surfaces.

Figure 4a,b show the reconstructed 3D shapes of the cylinder and the bottle surfaces. During the calculation, ROIs were first specified in the left sub-image of the cylinder and bottle surfaces with a grid step of 10 pixels, respectively. Then, the regularly spaced calculation points within the ROIs were tracked in the right sub-images to determine their disparity data with a subset size of 41 × 41 pixels. Finally, 3D coordinates of these points of interest were determined based on the calibrated extrinsic and intrinsic parameters and the disparity data. For each image, the calculation time was estimated as 0.8 s on the computer (CPU: Core i7-6700HQ, Intel, California, CA, USA). It is clearly observed that the reconstructed surface shapes of the cylinder and bottle surfaces are well in accordance with the real surface profiles. By fitting the 3D coordinates of the cylinder surface using the least square fit method, the diameter of the cylinder was estimated as 101.84 mm. Compared with the physical size (100.20 mm) determined by the Vernier caliper, the relative errors are estimated as 1.64%, confirming the accuracy of the established smartphone-based stereo-DIC system for shape measurement.

### 4.2. In-Plane and Out-of-Plane Displacement Measurement

**Figure 5:**
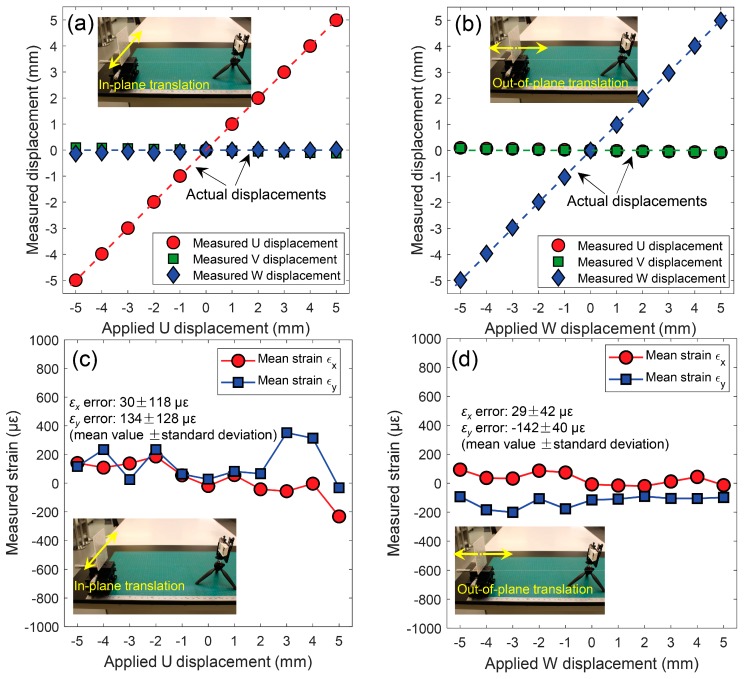
Measured *U*, *V* and *W* displacements (unit: mm) as a function of the applied displacements for (**a**) in-plane translation tests along the *X* direction and (**b**) out-of-plane translation tests along the *Z* direction. Measured horizontal and vertical strains (unit: με) as a function of the applied displacements for (**c**) in-plane translation tests along the *X* direction and (**d**) out-of-plane translation tests along the *Z* direction.

By processing the images recorded during the in-plane and out-of-plane translation tests, full-field 3D displacements within the specified ROI were retrieved with similar calculation parameters. The measured displacements of regularly spaced calculation points were then averaged and compared with the applied ones. Figure 5a,b show the mean values of the measured 3D displacements (*U*, *V*, *W*) as a function of the applied displacements for the in-plane and out-of-plane translation tests, respectively. As clearly presented in these two figures, the measured *X*-directional (i.e., *U*-) displacement for in-plane translation tests and *Z*-directional (i.e., *W*-) displacements for out-of-plane translation tests are in perfect agreement with the prescribed ones, while the other two directional displacements are near zero since no external displacement was applied in these two directions. The standard deviations of U and V displacements are less than 0.01 mm for both in-plane and out-of-plane translations, while those of W displacements are larger and determined as around 0.03 mm and 0.08 mm. The differences between the standard deviations of in-plane (*U* and *V*) and out-of-plane (*W*) displacements are highly correlated with the camera parameters. By differencing the measured full-field displacements, the full-field strain fields were obtained. These strain fields were also averaged and taken as the strain value for each translation, and then plotted in Figure 5c,d. Since no external load was applied on the specimen, the real strains should be zero for each translation, and the measured strains were seen as the strain errors. As shown in Figure 5c,d, the determined strain errors fluctuate around zero. Note that the standard deviations of strain fields are in the 195~350 με range. Compared with the strain errors (usually considered to be about 50 με) of a commercial system, those of smartphone-based stereo-DIC system are slightly larger but acceptable.

### 4.3. Stability Analysis of the Smartphone-Based Stereo-DIC System

#### 4.3.1. Thermal Variation and Virtual Strain Measurement

**Figure 6:**
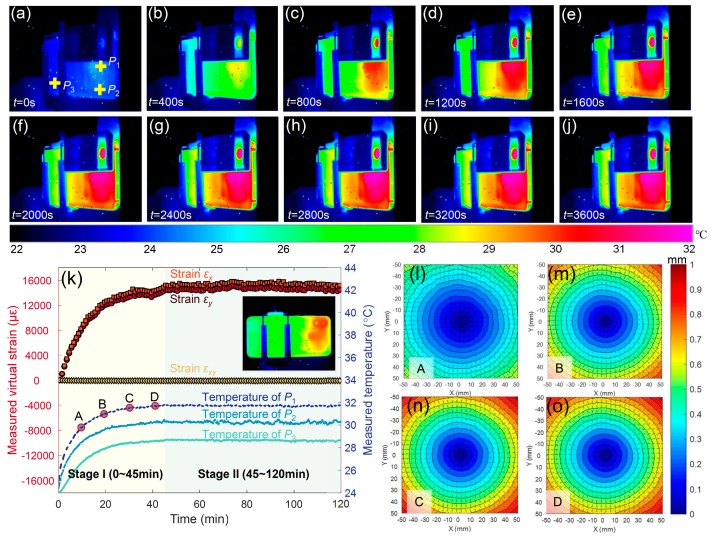
Temperature maps of the smartphone back surface monitored by the thermal camera during the static test: (**a**) *t* = 0 s, (**b**) *t* = 400 s, (**c**) *t* = 800 s, (**d**) *t* = 1200 s, (**e**) *t* = 1600 s, (**f**) *t* = 2000 s, (**g**) *t* = 2400 s, (**h**) *t* = 2800 s, (**i**) *t* = 3200 s and (**j**) *t* = 3600 s. (**k**) Measured virtual strains (unit: με) and temperature variations (unit: °C) of *P*_1,_ *P*_2_ and *P*_3_ as a function of the test time, (**l**–**o**) radial displacement vector superimposed on the radial contours at 10, 20, 30 and 40 min.

Figure 6 shows temperature maps of the smartphone back-side monitored by the thermal camera during the static test. As shown in these figures, there is a clear warm-up effect on the smartphone surface, which was caused by the smartphone self-heating. In particular, the temperatures near the camera are higher than those away from the camera. As the temperature distributions around the camera were covered by the optical attachment, a temperature map of the smartphone without the optical attachment, which was acquired in another test, is presented in the insert image of Figure 6k. To quantitatively show the temperature variations, the temperature histories of three points (*P*_1_, *P*_2_ and *P*_3_), as shown in Figure 6a on the smartphone back surface were extracted and plotted in Figure 6k, demonstrating that the smartphone experienced a warm-up stage (stage I, from the beginning to 45 min) followed by a thermal equilibrium stage (stage II, from 45 min to the end). The maximum temperature increase of *P*_1_ is about 8 °C, while those away from the smartphone camera are slightly smaller.

By processing the images recorded in the test, the full-field displacements and strains were retrieved. Since the smartphone and the test plate were both kept static and the indoor temperature was stable, the measured displacements and strains on the specimen surface can thus be regarded as the measuring errors caused by system instabilities, such as the structural change due to smartphone self-heating. Figure 6k shows the averaged strain errors (ε*_x_*, ε*_y_*, and ε*_xy_*) for each image recorded in the stationary test as a function of the recording time. Clearly, the virtual strain–time curves of ε*_x_* and ε*_y_* show a strong positive correlation with the temperature variations of the smartphone, while the shear strains (ε*_xy_*) are almost zero. Moreover, the maximum virtual strains due to the self-heating in the smartphone-based stereo-DIC system are much larger than those in a conventional stereo-DIC system [38]. Figure 6l–o show the measured radial displacement vectors superimposed on the radial contours at 10, 20, 30 and 40 min (corresponding the points A, B, C and D in Figure 6k), respectively. From the non-zero strains and concentrically spaced radial contour lines clearly shown in Figure 6l–o, it seems that the plate had undergone a nearly uniform thermal expansion, which is inconsistent with the real situation (the real deformation should be zero). Considering the positive correlation between the virtual strains and temperature variations (the test sample, the optical attachment and the smartphone were kept stable), the reason for the measuring errors can only be attributed to the changes of positions and orientations of the smartphone camera structures due to thermal expansion of the mechanical components in the camera.

#### 4.3.2. Modeling of Camera Self-Heating on the Single-Camera Stereo-DIC System

To explain the source of the large displacement and strain errors, a detailed analysis is necessary. As introduced before, the calculation of 3D displacements and strains rely on the reconstructed 3D coordinates, which were determined by the calibrated camera parameters (including the intrinsic and extrinsic parameters) and disparities between the two projection points in the left and right images according to triangulation principle. Ideally, the camera parameters and disparities should be constant in a static test where the cameras and the test sample were all kept stable. However, in the real test, the positions and orientations of the camera lens and sensor may be changed due to self-heating, and these changes will further lead to slight alterations in the positions of the two projection points. As a consequence, the disparities between the two projection points in the left and right images may also be altered, while the calibration parameters were regarded as constants during the calculation process although the real camera parameters changed over time due to the camera warming up. With the changing disparities and constant camera parameters, the reconstructed 3D coordinates will not be constant for the static test. To find the connections between the disparity variations and the inner changes of the smartphone camera, the movement or deformation of the smartphone camera due to the warm-up effect should be modeled. As the smartphone camera is highly integrated into the smartphone, it is very difficult to monitor the changes of the camera sensor and lens using the existing techniques, and we have adopted the existing camera deformation models to evaluate the movement or deformation of the smartphone camera components. 

**Figure 7:**
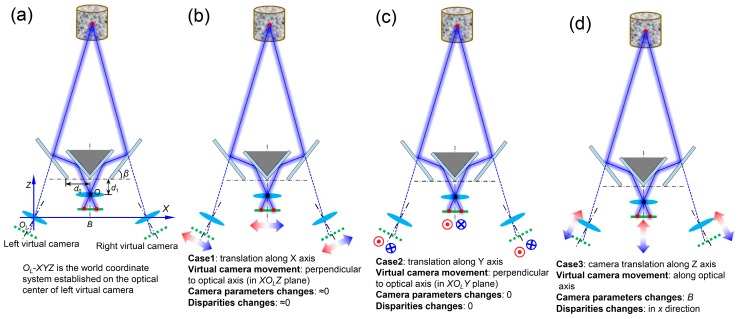
(**a**) Schematic diagram of the smartphone-based stereo-DIC system and (**b**–**d**) the three most possible movements of the smartphone.

In literature, efforts have been devoted to the modeling of the camera movement due to the warm-up effect. For example, a translation model [39,40] which assumes that only translations of the camera need to be considered and the intrinsic parameters remain constant was first used. Later, this model was shown to be inaccurate in some cases as the translations of the camera sensor and lens were not identical due to the different thermal conductivities in the sensor and lens components [41]. However, considering the small and highly-compact structure of smartphone camera, it is still reasonable to model the camera movement using a rigid-body translation model. Figure 7a shows the schematic diagram of the smartphone-based stereo-DIC system with its baseline distance estimated as [42] *B* = 4*d*_0_sin^2^*β* − 2*d*_1_cos2*β*, where *β* is the inclination of the outside mirror, *d*_0_ and *d*_1_ are the distances shown in Figure 7a. Note that *β* and *d*_0_ can be seen as constants as the mirrors frame is almost unaffected by temperature. Thus, the distance *d*_1_ is the only factor that influences the baseline distance. According to the rigid-body translation assumption, the three most possible movements of the smartphone camera are presented in Figure 7b–d, respectively. Note that the movements of the two virtual cameras are totally symmetric, which leads to a simpler analysis than a conventional two-camera based stereo-DIC system (for which the heating can differ from one camera to another). As the translations along *X* (Figure 7b) and *Y* (Figure 7c) directions will almost not change the baseline distance and angles of the optical axes of the two virtual cameras, the disparities of the measurement points will remain constant and only rigid-body motions will be added to the measured results. However, for the translation along the *Z* direction (Figure 7d), the baseline distance will change according to the aforementioned equation, which further will lead to a change in the disparities of the two projection points. Therefore, it can be concluded that only the translation along the *Z* direction will greatly change the imaging model of the single-camera stereo-DIC, while those in the other two directions will mostly introduce rigid-body motions to the two projection points.

**Figure 8:**
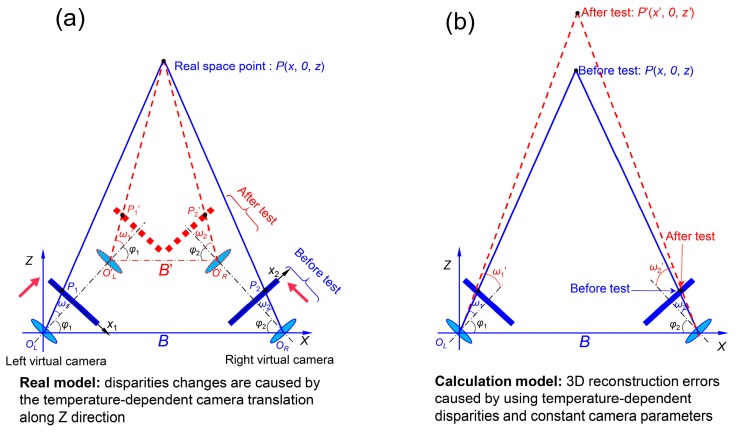
(**a**) A real model to explain how the disparities of the two projection points change during the test; (**b**) a calculation model to explain how the reconstruction errors are introduced during the calculation process.

To better understand the errors in the single-camera stereo-DIC system due to the camera warm-up effect, here we give two models, including a real model and a calculation model, to explain how the disparities of the two projection points change during the real test and how the reconstruction errors are introduced during the calculation process, respectively. As shown in Figure 8a, assuming the real camera only has a temperature-introduced translation along the positive *Z* direction, the two virtual cameras will move forward along their optical axes according to symmetric relationships in the single-camera stereo-DIC system. Obviously, the two projection points of a real space point (in *XOZ* plane) will move outward, which means a decrease in the *x* coordinate of *P*_1_ and an increase of the *x* coordinate in *P*_2_. As a result, the disparity (i.e., the difference in the *x* coordinates) of these two projection points in the *x* direction will increase due to the camera translation. During the calculation, the calibrated camera intrinsic and extrinsic parameters were used to build a world coordinate system, as shown in Figure 8b. As all the parameters are fixed for all the images recorded in the self-heating test, the temperature-dependent disparities undoubtedly will result in temperature-dependent 3D coordinates, and these varying 3D coordinates further lead to temperature-dependent displacements and strains.

#### 4.3.3. Verification of the Analysis

**Figure 9:**
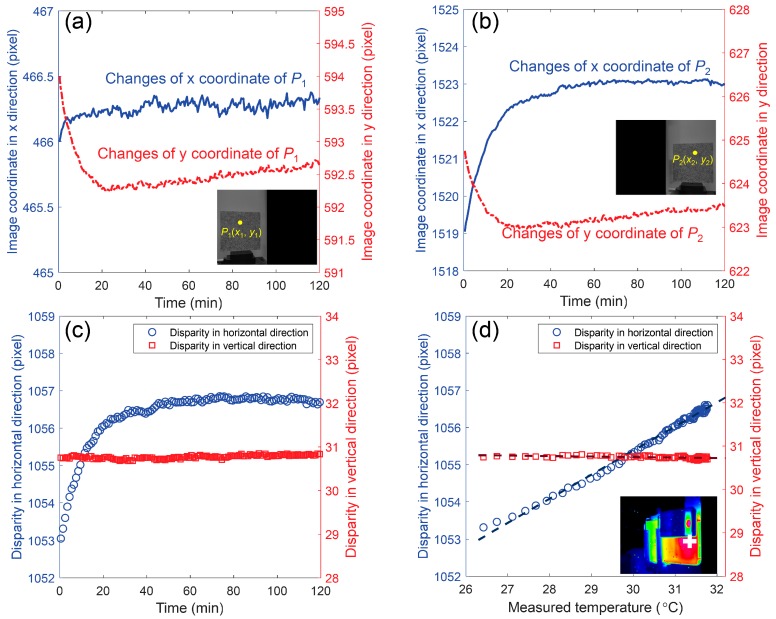
The variations of the image coordinates of the two projection points (**a**) *P*_1_ and (**b**) *P*_2_ as a function of the test time, and the disparities of point *P* (i.e., the differences in image coordinates between *P*_1_ and *P*_2_) as a function of (**c**) the test time and (**d**) the measured temperature.

Figure 9a,b show the variations of the image coordinates of the two projection points (*P*_1_, *P*_2_) as a function of the test time. It can be seen that all the coordinates present a similar trend (positive for *x* and negative for *y*) with the temperature variation and the amplitudes of the variations in *y* coordinates are almost equal, which can be well explained by the movement of the camera along the *Y* (Figure 7c, perpendicular to *XZ* plane) direction. However, the variation of the *x* coordinate of *P*_2_ is larger than that of *P*_1_ in amplitude, which ideally should be equal if only translation along the Z direction existed. According to a previous analysis, this difference can be attributed to a joint influence of translations along *X* and *Z* directions. The disparity variations between the two projection points are plotted in Figure 9c. It is clear that the disparities in the vertical direction are nearly constant during the test, while those in the horizontal direction present a similar trend with the temperature variations of the smartphone back surface. To find the connection between the disparity variation and the temperature rise, the disparities between the two projection points were plotted as a function of the temperature of a point on the smartphone back side and are presented in Figure 9d. From this figure, it is not difficult to see that the disparities in the *x* direction have a linear relationship with the temperature variation of the smartphone. 

**Figure 10:**
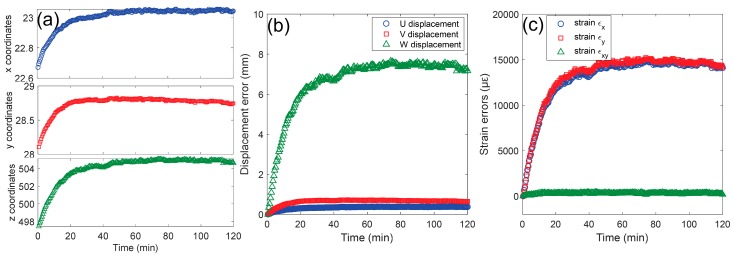
The measured (**a**) 3D coordinates (unit: mm), (**b**) displacements (unit: mm) and (**c**) strains (unit: με) of a point *P* as a function of the test time.

According to the triangulation principle shown in Figure 8b, the temperature-dependent disparities will further result in temperature-dependent 3D coordinates, displacements and strains as the camera parameters are regarded as constant during the analysis. Figure 10a–c show the measured 3D coordinates, displacements and strains of a point *P* as a function of the test time using the temperature-dependent disparities. Except the shear strain shown in Figure 9c, all the other measured values indicate similar trends to the measured temperature, and these values will vary from point to point on the specimen surface. In real tests, these displacements and strain errors may exceed the real deformation on the specimen surface and are thus unacceptable. To eliminate these errors and realize high-accuracy measurement as much as possible, the following three approaches can be employed [43]: (1) the preheating of the digital camera before image acquisition; (2) error correction using a pre-established artificial deformation-temperature/time curve; (3) deformation compensation using a reference sample placed near the test specimen. 

## 5. Applications of the Smartphone-Based Stereo-DIC System to 2D and 3D Deformation Measurement

### 5.1. Tensile Test of CFRP Specimen

**Figure 11:**
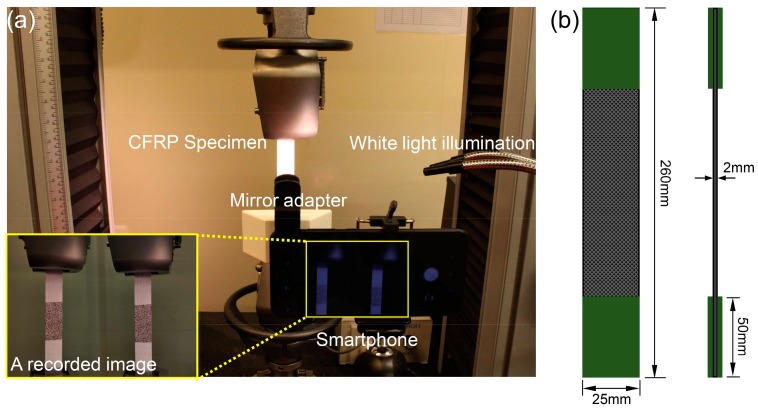
(**a**) Experimental set-up used to examine the mechanical properties of the carbon fiber-reinforced plastics (CFRP) specimens, with a captured image using the established imaging system shown in the insert image. (**b**) Schematic diagram of the CFRP specimen.

To investigate the effectiveness and accuracy of the smartphone-based stereo-DIC system in real deformation measurement, uniaxial tensile tests were conducted. The test specimens were rectangular CFRP specimens (250 mm × 25 mm × 2 mm in size), and the substrate materials of the specimen were unidirectional carbon fiber pre-pregs composed of toughened epoxy resin and carbon fibers (HexPly T700/M21, Hexcel, Stamford, CT, USA), with a nominal fiber volume of 57%. Unidirectional laminates ([0°]_8_) were fabricated by compression molding. The elastic modulus and Poisson’s ratio of the CFRP were determined as 135 ± 6 Mpa and 0.29 ± 0.01 using strain gauges, respectively. Before the test, the specimens were speckled and clamped using the universal tensile machine (Instron 5882, Instron, Norwood, MA, USA) with a 100kN load cell, as shown Figure 11. The imaging system was placed in front of the test sample with the optical axis of the camera approximately normal to the specimen surface. To eliminate the errors due to the smartphone camera self-heating, the smartphone-based stereo-DIC system was preheated for 45 min by recording images every 30 s. Then, an image of the specimen was recorded as the reference image and the smartphone-based stereo imaging system was carefully calibrated using a regular calibration target. Subsequently, the CFRP specimen was loaded in increments of 5 kN per load stage using the load control mode with a loading speed of 10 kN/min. Once the applied load reached the preset values, the tensile machine stopped loading and held the specimen in force control mode, and then a digital image was recorded for the strain measurement. The maximum tensile load applied was 50 kN, which is below the yield force of the specimen. The same loading process was repeated for the other two specimens.

**Figure 12:**
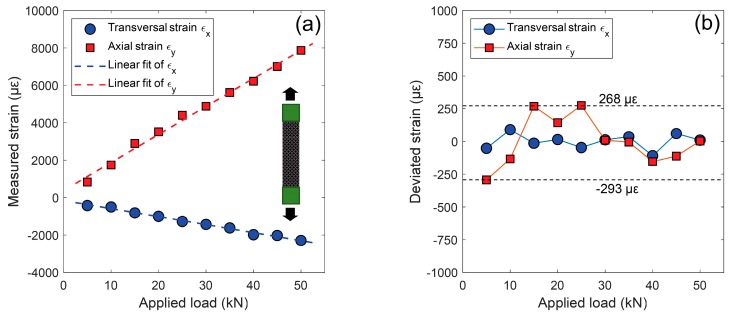
(**a**) Measured transversal (*ε_x_*) and longitudinal (*ε_y_*) strains (unit: με) as a function of the applied loads and (**b**) deviated strains between the measured strains and fitted strains.

By processing these recorded images using the aforementioned approach, full-field displacements and strain fields on the specimen surface were retrieved and used to determine the mechanical properties of the CFRP specimen. Figure 12a shows the average transversal and longitudinal strains measured by the smartphone stereo-DIC system at various loading stages. It is seen that the measured strains were proportional to the applied loads. Taking the fitted strains as references, the strain differences between the measured strains and the fitted ones were plotted in Figure 12b. The standard deviations of these strain differences were estimated as 58 με and 184 με for the transversal and longitudinal strains. Compared with a professional stereo-DIC system, the strain variations are slightly larger but acceptable for a smartphone-based system. This is because the smartphone stereo-DIC system is more sensitive to the matching uncertainties and environmental disturbance (e.g., temperature variation) than the conventional stereo-DIC system. The underlying reason for this difference may be attributed to the differences in camera parameters, such as the baseline distance and focal length. The elastic modulus and Poisson’s ratio of the test specimen were then estimated using the following equation,

(1) $$\begin{array}{c} E=\frac{F}{A\cdot \varepsilon_{y}}=\frac{1}{A\cdot \varepsilon_{y}/F}\\ \nu =-\frac{\varepsilon_{x}}{\varepsilon_{y}}=-\frac{\varepsilon_{x}/F}{\varepsilon_{y}/F} \end{array}$$

where *ε_x_*/*F* and *ε_y_*/*F* are the slopes of the two fitting lines, and A is the cross-sectional area of the specimen. Table 2 summarizes the elastic modulus and Poisson’s ratios measured by the established system for the three tensile tests. The mean values of the elastic modulus and Poisson’s ratio are 133.59 GPa and 0.293, respectively. The differences between the mechanical properties measured by the strain gauge and those measured by the smartphone are very small (1.04% difference in elastic modulus and 1.03% difference in Poisson’s ratio, which can be fully attributed to the different scale at which the parameters are identified due to the different gauge lengths), confirming the accuracy of the smartphone-based system in mechanical testing.

### 5.2. Three-Dimensional Deformation Measurement of a Deflating Ball

**Figure 13:**
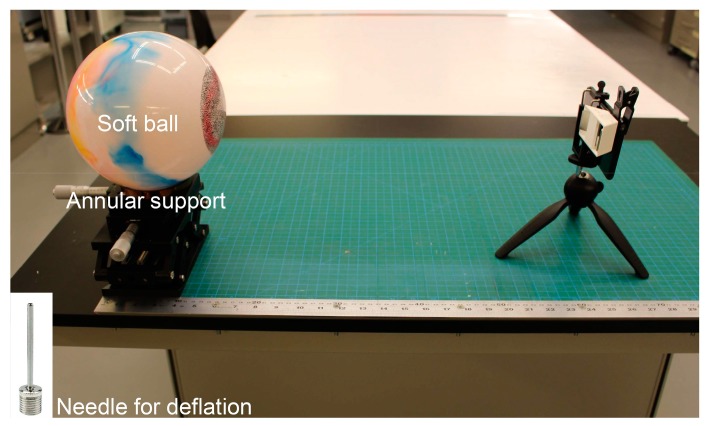
Experimental set-up for the 3D deformation measurement of a deflating ball. The insert image is the needle for deflation during the test.

A deformation measurement test of a deflating ball was also conducted to verify the effectiveness of the smartphone-based stereo-DIC system in real 3D deformation measurement. Figure 13 shows the experimental set-up for the 3D deformation measurement. The test object was a hollow soft ball (bought from a supermarket) with a physical size measured as 198.2 mm in diameter (the ball was clamped using two flat plates and then the distance between the two plates was measured using a Vernier clipper; this process was repeated three times from different directions). Before the test, the ball was speckled using a black marker pen and placed on an annular support to keep it stable. The smartphone-based stereo-DIC system was placed before the ball and also preheated for 45 min to eliminate the errors due to the smartphone camera self-heating. During the test, a reference image was first recorded as the reference image, and then deformed images were captured every five seconds after inserting a needle into the ball. These images, together with the calibration images, were used to reconstruct the 3D shapes on the ball surface.

**Figure 14:**
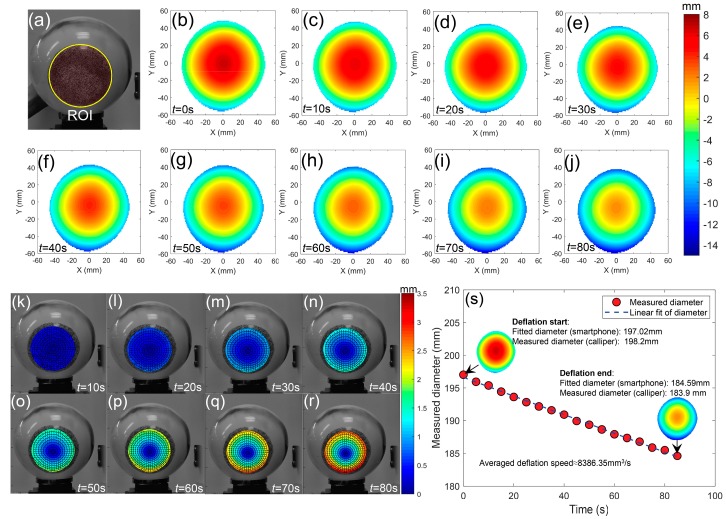
(**a**) The region of interest during the calculation and (**b**–**j**) the reconstructed 3D shapes on the ball surface at different time steps, (**k**–**r**) radial displacement vector superimposed on the radial contours on the ball surface at different time steps, (**s**) the fitted diameters as a function of time.

By analyzing these images using the method introduced before, the 3D shape, displacement and deformation within the ROI (shown in Figure 14a) at different time steps were obtained. Figure 14b–j show the 3D shapes on the ball surface at the corresponding moment. From these profiles, the whole deformation process on the ball surface due to the deflation were clearly observed. To better present the deformation of the ball, the in-plane radial displacement fields of different times are given in Figure 14k–r. As indicated in these figures, an apparent shrink is found on the ball surface due to the deflation and amount of contraction increase with time. The maximum in-plane contraction was measured as about 3 mm at the 80th second. By fitting the reconstructed 3D coordinates of the ball surface using the least square method, diameters of the ball at different times were determined and plotted as a function of test time, as shown in Figure 14s. The measured diameters present an almost uniform downward trend during the deflation process. By comparing the fitted diameters before and after deflation (197.02 mm and 184.59 mm) with the directly measured ones (198.2 mm and 183.9 mm) using a Vernier clipper, the relative errors were determined as 0.6% and 0.4%, validating the accuracy of the smartphone-based stereo-DIC system in real 3D deformation measurement.

## 6. Conclusions

In this paper, we established an ultra-portable and cost-effective stereo-DIC system for accurate 3D shape, displacement and deformation measurements using a smartphone and an optical attachment. Compared with existing two-camera or single-camera stereo-DIC systems, the smartphone-based stereo-DIC system is simpler, as well as being portable and cost-effective, thus demonstrating a great potential in resource-limited and field settings. The effectiveness and accuracy of this system in 3D shape and displacement measurements were first verified through shape measurements and in-plane and out-of-plane translation tests. The stability of the smartphone-based stereo-DIC system was then studied in a static test with a maximum virtual strain measured as approximately 15,000 με. The source of the measuring errors in the single-camera stereo-DIC system are well explained here for the first time. Finally, the smartphone-based system was prone to be accurate in the material properties determination of a CFRP specimen with a comparable accuracy to the strain gauge, as well as for 3D deformation measurements of a deflating ball after a simple preheating process. 

More importantly, except for high-resolution digital cameras, there are various sensors (e.g., GPU, wireless transmission, gravity sensor, acceleration sensor, gyroscope and GPS) in a smartphone. This superior performance makes smartphones an ideal platform for conducting various scientific research, which would also positively impact research and educational efforts in developing countries and resource-limited institutions, helping the popularization of advanced scientific instruments and measurement tools. Besides this, as the recorded images are exported to the computer for further processing, developing an app integrated with the smartphone is therefore promising for smartphone-based stereo-DIC systems. With its superior computational performance, a smartphone can even realize high-speed, real-time 3D displacement tracking once the thermal errors are thoroughly eliminated.

## Figures and Tables

**Table 1:** The calibrated intrinsic and extrinsic parameters for the smartphone-based stereo-DIC system.

	Intrinsic Parameters		Extrinsic Parameters	
Left camera	*c_x_* (pixel)	1034.76	*α* (deg)	−1.305
	*c_y_* (pixel)	764.58	*β* (deg)	27.878
	*f_x_* (pixel)	2642.17	*γ* (deg)	−0.092
	*f_y_* (pixel)	2647.80	*t_x_* (mm)	−47.169
	*k*_1_	0.119	*t_y_* (mm)	0.039
Right camera	*c_x_* (pixel)	1024.21	*t_z_* (mm)	12.219
	*c_y_* (pixel)	746.63	Baseline (mm)	48.73
	*f_x_* (pixel)	2658.91		
	*f_y_* (pixel)	2661.20		
	*k*_1_	0.142		

**Table 2:** The elastic modulus and Poisson’s ratio measured by the smartphone-based stereo-DIC system.

Test No.	Elasticity Modulus (GPa)	Poisson’s Ratio
Test 1	132.80	0.274
Test 2	135.95	0.320
Test 3	132.04	0.286
Mean vale	133.59	0.293
